# Steroid resistance in COPD is associated with impaired molecular chaperone Hsp90 expression by pro-inflammatory lymphocytes

**DOI:** 10.1186/s12931-016-0450-4

**Published:** 2016-10-21

**Authors:** Greg Hodge, Eugene Roscioli, Hubertus Jersmann, Hai B. Tran, Mark Holmes, Paul N. Reynolds, Sandra Hodge

**Affiliations:** 1 Lung Research, Hanson Institute and Department of Thoracic Medicine, Royal Adelaide Hospital, Adelaide, South Australia Australia; 2 Department of Medicine, University of Adelaide, Adelaide, South Australia Australia

**Keywords:** Lymphocyte senescence, COPD, Hsp90, CD28nullCD8+ T and NKT-like cells, IFNγ and TNFα

## Abstract

**Background:**

Corticosteroid resistance is a major barrier to effective treatment of COPD. We have shown that the resistance is associated with decreased expression of glucocorticoid receptor (GCR) by senescent CD28nullCD8+ pro-inflammatory lymphocytes in peripheral blood of COPD patients. GCR must be bound to molecular chaperones heat shock proteins (Hsp) 70 and Hsp90 to acquire a high-affinity steroid binding conformation, and traffic to the nucleus. We hypothesized a loss of Hsp70/90 from these lymphocytes may further contribute to steroid resistance in COPD.

**Methods:**

Blood was collected from COPD (*n* = 10) and aged-matched controls (*n* = 10). To assess response to steroids, cytotoxic mediators, intracellular pro-inflammatory cytokines, CD28, GCR, Hsp70 and Hsp90 were determined in T and NKT-like cells in the presence of ± 10 μM prednisolone and 2.5 ng/mL cyclosporine A (binds to GCR-Hsp70/90 complex) using flow cytometry, western blot and fluorescence microscopy.

**Results:**

A loss of expression of Hsp90 and GCR from CD28null CD8+ T and NKT-like cells in COPD was noted (Hsp70 unchanged). Loss of Hsp90 expression correlated with the percentage of CD28null CD8+ T and NKT-like cells producing IFNγ or TNFα in all subjects (eg, COPD: *R* = −0.763, *p* = 0.007 for T-cell IFNγ). Up-regulation of Hsp90 and associated decrease in pro-inflammatory cytokine production was found in CD28nullCD8+ T and NKT-like cells in the presence of 10 μM prednisolone and 2.5 ng/mL cyclosporine A.

**Conclusions:**

Loss of Hsp90 from cytotoxic/pro-inflammatory CD28nullCD8+ T and NKT-like cells could contribute to steroid resistance in COPD. Combination prednisolone and low-dose cyclosporine A therapy inhibits these pro-inflammatory cells and may reduce systemic inflammation in COPD.

## Background

Chronic obstructive pulmonary disease (COPD) is a leading cause of death worldwide and existing treatments, such as anti-inflammatory corticosteroids, have no proven disease modifying effect [1] although a reduction in exacerbation rates and improved health status has been reported [2]. The mechanisms underlying this resistance are largely unknown, particularly in lymphocytes [2]. It has been suggested that CD8+ T cells are the central regulator of the inflammatory network in COPD [3]. CD8+ T cell deficient mice had a blunted inflammatory responses and did not develop emphysema when exposed to long-term cigarette smoke [3]. We have reported increased production of pro-inflammatory cytokines and expression of cytotoxic mediators granzyme b and perforin in CD8+ T cells in the peripheral blood and lungs [4] of current and ex-smoker COPD patients compared to healthy smokers and never-smokers [5].

Our research has focused on identifying the lymphocyte subset/s resistant to current therapeutics and we have made several important discoveries. We have shown that COPD is associated with increased CD28nullCD8+ senescent cells in the peripheral blood of both current and ex-smoker COPD subjects, and showed these cells are more cytotoxic/pro-inflammatory than CD8 + CD28+ cells [6]. NKT-like and NK cells were increased in bronchoalveolar lavage of COPD patients and associated with increased cytotoxicity [7]. In this regard, CD8 + CD28null NKT-like cells have been shown to be more pro-inflammatory and cytotoxic than CD8 + CD28+ NKT-like cells in other pro-inflammatory lung diseases [8].

CD28nullCD8+ pro-inflammatory lymphocytes have decreased levels of histone deacetylase 2 (a nuclear enzyme required by corticosteroids to switch off activated inflammatory genes) [9] and reduced levels of glucocorticoid receptor (GCR) [10].

GCR must be bound to the molecular chaperones heat shock proteins (Hsp) 70 and Hsp90 to acquire a high-affinity steroid binding conformation, and traffic to the nucleus [11]. We hypothesized a loss of Hsp70/90 from these lymphocytes may contribute to steroid resistance in COPD.

To investigate this hypothesis, we determined whether peripheral blood CD28null T cells and NKT-like cells (particularly CD8+) from COPD patients express reduced levels of Hsp70 and Hsp90 and whether loss of these molecular chaperones is associated with a lack of suppression of cytotoxic mediators or pro-inflammatory cytokines produced in response to steroid treatment. The immunosuppressant, cyclopsorin A (CsA) binds to the GCR-Hsp90 complex but not Hsp70 [12]. We therefore also investigated the effect of CsA in combination with the corticosteroid prednisolone, on Hsp70/90 and associated pro-inflammatory cytokine expression by lymphocyte subsets.

## Methods

### Patient and control groups

COPD volunteers were specifically recruited for the study and informed consent obtained. There was no exacerbation of COPD for 6 weeks prior. Subjects with other co-existing lung disease or malignancy or aged greater than 75y were excluded. Ethics approval was obtained from the Royal Adelaide Hospital and the experiments were conducted with the understanding and the written consent of each participant. COPD was diagnosed using the GOLD criteria with clinical correlation (mild COPD: FEV1/FVC < 70 % but FEV1 ≥ 80 % predicted; moderate COPD FEV1 50 % ≤ 80 % predicted, severe COPD FEV1 30 % ≤ 50 % predicted, very severe COPD FEV1 < 30 % predicted) [13]. Blood was collected from 10 patients with COPD (Table 1) all of whom were ex-smokers (at least one year) with an average of 37 pack years. No patients were receiving oral corticosteroids although 8/10 patients were taking inhaled steroids.

**Table 1:** Demographic details of the COPD and control group

Subjects	Controls	COPD
No. of subjects	10	10
Age (years)	49 (41–57)	58 (42–64)*
FEV1, % pred	108.4 (91–110)	60.1 (44–96)*
FEV1/% FVC	96 (84–108)	58 (43–73)*
Male/Female	8/6	6/4

Data showing median (range)

Abbreviations: *FEV*1 forced expiratory volume in 1 s, *FVC* forced vital capacity; *p < 0.05 compared to controls

Blood was also obtained from 10 aged-matched non-smoking volunteers (Table 1) with normal lung function. These were healthy, recruited volunteers with no history of airways disease. All subjects underwent spirometry as part of their routine clinical assessment. Venous blood was collected into 10 U/mL preservative free sodium heparin (DBL, Sydney, Australia), and maintained at 4 °C until processing within 4 h. All patients were submitted to the same protocol and analysis performed retrospectively.

### Hsp70, granzyme b and perforin expression in T and NKT-like cell subsets

Hsp70 was constitutively expressed in lymphocyte subsets, however stimulation of cells was required for expression of Hsp90 as previously shown, hence mimicking Hsp90 induction following stress response [14]. To determine expression of Hsp70 and cytotoxic mediators granzyme b and peforin in CD8+ and CD8-T and NKT-like cells, aliquots of blood were added to FACS tubes and red blood cells were lysed using FACSLyse (BD Biosciences, Sydney, Australia) as described previously [9]. After 10 min, tubes were centrifuged at 300 g for 5 min, supernatant discarded and leucocytes permeabilised using FACSPerm (BD) as previously reported [9]. Cells were then washed with wash buffer (0.5 % BSA in Isoflow (Beckman Coulter, Sydney, Australia)), and appropriately diluted monoclonal antibodies (Mabs) added as previously reported [15]: anti-Hsp70 AF488 (clone W27, Biolegend, Sydney, Australia), perforin PE (BD), CD3 perCP.CY5.5 (BD), CD28 PECY7 (BD), CD56 APC (Beckman Coulter), CD8 APC.CY7 (BD), granzyme B V450 (BD) and CD45 V500 (BD). After washing cells in wash buffer, centrifugation and decanting, cells were analyzed within 1 h on a FACSCanto II flow cytometer using FACSDiva software (BD). Samples were analyzed by gating lymphocytes using CD45 staining versus side scatter (SSC). A minimum of 350,000 low SSC events were acquired in list-mode format for analysis. T cells were identified as CD3 + CD56-CD45+ and NKT-like cells identified as CD3 + CD56+ CD45+ low FSC/SSC events [9].

### Hsp90, GCR and intracellular cytokine expression in T and NKT-like cell subsets

To determine co-expression of Hsp90 and GCR with intracellular cytokines in CD8+ and CD8-T and NKT-like cells, aliquots of blood were stimulated as previously reported [3] with phorbol myristate acetate (25 ng/mL) (Sigma, Sydney, Australia) and ionomycin (1 μg/mL) (Sigma) in the presence of brefeldin A (1 μg/mL) (Sigma) and the tubes incubated in a humidified 5 % CO_2_/95 % air atmosphere at 37 °C. The addition of brefeldin A had no effect on Hsp90 or GCR expression in these experiments (not shown). At 16 h, 100 μL 20 mM EDTA/PBS was added to the culture tubes, followed by vigorous vortexing for 20 s to remove adherent cells. Red blood cells were lysed and cells were permeabilised as described previously [3]. Two mL 0.5 % bovine serum albumin (Sigma/Aldrich, Sydney, Australia)/Isoflow (Beckman Coulter, Sydney, Australia) was then added and the tubes centrifuged at 300 × g for 5 min. After decanting the supernatant, Fc receptors were blocked with 10 μL human immunoglobulin (Intragam: CSL, Parkville, Australia) for 10 min in the dark at RT. Cells were stained with appropriately diluted anti-Hsp90 Mab (clone AC88, Abcam, Sydney, Australia) as described above. Following washing of cells, cells were stained with rat anti-mouse IgG1 PE (BD) for 15 min in the dark at RT. Cells were stained with appropriately diluted anti-GCR Mab (clone 5E4, Serotec, Sydney, Australia) as described previously [10]. Cells were then washed and stained with rat anti-mouse IgG1 V450 (BD) for 15 min in the dark at RT. Cells were further washed and appropriately diluted Mabs to IFNγ FITC, TNFα FITC (BD), CD3 perCP.CY5.5 (BD, Sydney, Australia), CD28 PECY7 (BD), CD56 APC (Beckman Coulter, Sydney, Australia), CD8 APC.CY7 (BD) and CD45 V500 (BD) added for 15 min in the dark at RT. Following a final wash, cells were analyzed within 1 h on a FACSCanto II flow cytometer using FACSDiva software (BD). Samples were analyzed by gating lymphocytes using CD45 staining versus side scatter (SSC). A minimum of 350,000 low SSC events were acquired in list-mode format for analysis. T cells were identified as CD3 + CD45+ and NKT-like cells identified as CD3 + CD56+ CD45+ low FSC/SSC events.

### Hsp90 expression in CD28+ and CD28null T cells by Western Blot

Expression of Hsp90 has not previously been validated using flow cytometry therefore confirmation of Hsp90 staining was determined using western blot and immunofluorescence. PBMC were isolated from blood of cohorts of control (*n* = 2) and COPD patients (*n* = 3) by standard density gradient centrifugation and cells re-suspended at 1 × 10^7^ mL in RPMI 1640 medium. Following stimulation as described above, 5 μL of appropriately diluted CD3 perCP.CY5.5 (BD), CD28 PE.CY7 (BD), CD56 APC (Beckman Coulter), CD8 APC.CY7 (BD) and CD45 V500 (BD) Mabs were added for 15 min in the dark at room temperature. Cells were washed and resuspended in 1 mL RPMI and CD28+ and CD28null, CD8+ and CD8-T cells were immediately sorted on a FACSAria flow cytometer (BD).

Equal numbers of sorted CD28+ and CD28 null T cells were lysed using M-Per mammalian cell protein lysis reagent with Halt® protease inhibitor cocktail (both Thermo Scientific, Victoria, Australia). Protein samples were quantified using the DC protein assay (Bio-Rad, Victoria, Australia), and 10 μg electrophoresed using Novex® 4–12 % gradient Bis-Tris denaturing gels (Life Technologies, Victoria, Australia) and electroblotted to Trans-Blot® Turbo nitrocellulose membrane (Bio-Rad). Membranes were blocked in 5 % diploma skim milk, washed, then incubated overnight at 4 °C with anti-human Hsp90 (1:2000), followed by a 1 h incubation at RT with horse radish peroxidase-labelled anti-mouse secondary antibody (R&D Systems, MN, USA). Chemiluminescent imaging was performed using the LAS-3000 platform, and histogram analysis performed using the Multigauge software package (both FugiFilm, Tokyo, Japan). Mouse-anti Human β-actin antibody (Sigma-Aldrich, MO, USA) was used to correct for loading error for histogram analyses.

### Immunoprecipitation

Immunoprecipitation, in conjunction with western analysis were used to confirm Hsp90 protein in complex with the GCR. Equal numbers of sorted CD28+ and CD28 null T-cells were isolated from a patient with COPD and were lysed in protein extraction buffer, in the presence of protease inhibitors (as above). Protein samples were precipitated using protein G Dynabeads® (Life Technologies, Victoria, Australia) labelled with GCR monoclonal antibody and the eluates were probed for Hsp90 using western analysis (as outlined above). Unlabeled bead controls were also incubated with protein samples to assess for nonspecific binding.

### Hsp90 expression in CD28+ and CD28null T cells by Fluorescent Microscopy

Fluorescence microscopy was used to validate Hsp90 staining. 1 × 10^3^ sorted CD28+ and CD28 null T cells (as described above) were added to a Cytospin 4 cytocentrifuge (ThermoFisher Scientific, Scorseby, Victoria, Australia) and centrifuged for 500 g for 5 min. Slides were air dried for 10 min and cells fixed with 2.5 % formalin in PBS for 10 min. Cytospins were treated with 1 % sodium dodecyl sulphate (SDS, Sigma Aldrich, Castle Hill, NSW, Australia) in PBS for 5 min, followed by 1 h incubation with a serum-free protein blocker (Dako A/S, Glostrup, Denmark), overnight incubation at 4 °C with 1/25 diluted Hsp90 monoclonal antibody (Serotec, Abacus ALS, Brisbane, Australia), then 1 h with AF594-conjugated donkey IgG F (ab’) 2 fragment polyclonal antibody to mouse IgG (Abcam, Sapphire Bioscience, Waterloo, NSW, Australia), and counterstained with DAPI (Sigma-Aldrich). Cells were washed between incubation with 0.01 M Tris-buffered saline pH 7.5, containing 0.05 % Tween-20. Immunofluorescence was detected and imaged with a Olympus IX73 fluorescence microscope (Olympus, Notting Hill, VIC, Australia). For quantitative analysis, cells from each cytospin were photographed under a 40× objective in 8 optical fields, selected in the DAPI channel for bias prevention, the mean fluorescence intensities measured then in the AF594 channel using the ImageJ software (NIH, Bethesda, MD, USA) as previously described [10].

### Effect of drugs on Hsp90, GCR and intracellular IFNγ and TNFα expression in T and NKT-like cell subsets

The immunosuppressant, CsA binds to the GCR-Hsp90 complex but not Hsp70 [11]. We therefore also investigated the effect of CsA in combination with the corticosteroid prednisolone on Hsp90 and associated pro-inflammatory cytokine expression by lymphocyte subsets. CsA is a Pgp-1 inhibitor and we have previously shown that pro-inflammatory cytokine production was significantly reduced in T and NKT-like cells in the presence of very low dose cyclosporine A (2.5 ng/mL) [16]. To determine the effect of these drugs on Hsp90 expression in pro-inflammatory T and NKT-like cells, aliquots of blood were mixed in 10 mL sterile tubes with equal volume of RPMI 10 % FCS and incubated with ± 1 μM prednisolone ± 2.5 ng/mL CsA and the tubes incubated in a humidified 5 % CO_2_/95 % air atmosphere at 37 °C for 24 h. Blood cultures were then stimulated as for intracellular cytokine production as described above for 16 h. Aliquots of blood cultures were then processed as for intracellular cytokines and HSP90, IFNγ and TNFα expression as described above.

### Statistical analysis

Statistical analysis was performed using the Wilcoxon sign rank test. For T-cell subsets (CD28null/CD8+/CD3+/CD56−/CD45+/TNFα+/IFNγ+), a sample size of *n* = 10 allowed a power of 98–99.5 % for analysis. Variance was estimated from our previous studies [4–7]. Correlations were performed using Spearman Rho correlation tests. SPSS software was applied and differences between groups of *p* < 0.05 considered significant.

## Results

### Increased CD28null CD8+ T and NKT-like cells in COPD patients

There was a significant increase in CD28nullCD8+ T cells in patients with COPD compared with healthy controls (*p* < 0.05), but no change in CD28nullCD8- T cells (CD28nullCD8+ T: 56 ± 7.7 (32 ± 7.5); CD28nullCD8- T: 6.9 ± 3.3 (6.1 ± 4.3) for COPD patients (controls) (median ± sem) consistent with our previous findings for CD28null T cells [6]. There was a significant increase in CD28nullCD8+ NKT-like cells in patients with COPD compared with healthy controls but no change in CD28nullCD8- NKT-like cells (CD28nullCD8+ NKT-like: 41 ± 6.8 (23 ± 6.6); CD28nullCD8- T: 8.6 ± 3.8 (7.6 ± 3.5) for COPD patients (controls)).

### Hsp70, perforin and granzyme B expression by CD28+ and CD28null T and NKT-like cells

A higher percentage of CD28nullCD8+ T cell and NKT-cells expressing perforin and granzyme b was found in COPD patients compared with control subjects (eg., 44 ± 12 (15 ± 13) *p* = .031; and 34 ± 11 (13 ± 9) *p* = .021 for the percentage of CD28null CD8+ T cells expressing granzyme b and perforin (median ± sem) from COPD patients (controls) respectively, consistent with a previous report [9]. There was no change in perforin or granzyme b expression in CD28 + CD8+ or CD28 + CD8-T and NKT-like cells from COPD or control groups (*p* > 0.05 for all) also consistent with a previous report [9].

There was no change in the percentage of CD28nullCD8+ T cell and NKT-cells expressing Hsp70 in COPD patients compared with control subjects (*p* > 0.05 for all) (Table 1). There was no change in Hsp70 expression between CD28+ or CD28null CD8+ or CD8-T or NKT-like cells between COPD patients or control subjects (*p* > 0.05 for all) (Table 2). The was no correlation between the percentage of any lymphocyte subset expressing Hsp70 and granzyme b or perforin (*p* > 0.05 for all).

**Table 2:** Percentage of lymphocyte subsets expressing Hsp70

	CD3 + CD8 + CD28null T-cells	CD3 + CD8 + CD28+ T-cells	CD3 + CD8 + CD56 + CD28null NKT-like cells	CD3 + CD8 + CD56 + CD28+ NKT-like cells
COPD	15.9 (4–31)	18.8 (3–35)	17.3 (4–36)	14.8 (3–39)
Control	19.7 (5–33)	17.3 (3–33)	15.9 (5–32)	15.9 (2–41)

Data showing median (range)

*p* > 0.05 for all data compared to controls

### Hsp90, GCR and intracellular cytokine expression in T and NKT-like cell subsets

A significant increase in the percentage of CD28nullCD8+ T and NKT-like cells producing IFNγ and TNFα compared with CD28 + CD8+ T and NKT-like cells was noted in COPD patients and control groups consistent with a previous report [9, 10] (data not shown).

**Fig. 1:**
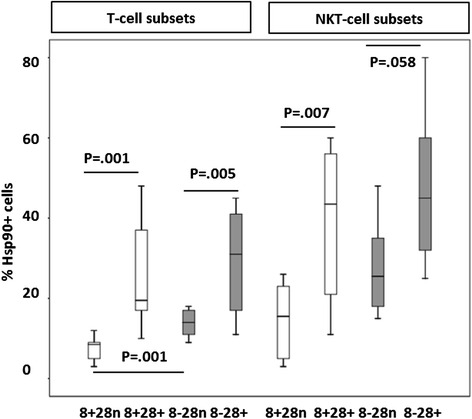
The percentage of CD28+ and CD28null CD8+ (clear bars) and CD8-T cells expressing Hsp90 in patients with COPD. There was a significant decrease in the percentage of CD8 + CD28null (8 + 28n) T and NKT-like cells expressing Hsp90 (clear bars) compared with CD8 + CD28+ and CD28 + CD8- T and NKT-like cells (*grey bars*) (trend for CD8-CD28 + NKT-like cells) and a decrease in Hsp90 expression in CD8 + CD28null compared with CD8-CD28+ T cells. Box plots present median ± 25th and 75th percentiles (solid box) with the 10th and 90th percentiles shown by whiskers outside the box.*significantly (*p* < 0.05) decreased expression compared to controls

For both COPD groups and controls, a significantly lower percentage of CD28nullCD8+ T and NKT-like cells expressing Hsp90 was found, compared with CD28+ T and NKT-like cells (data for T cell and NKT-like cell subsets from COPD group shown in Fig. 1) (data for controls not shown).

A significantly lower percentage of CD28nullCD8+ T and NKT-like cells expressing GCR was found, compared with CD28+ T and NKT-like cells from all groups tested. This is consistent with previous findings [14] (data not shown).

There was no difference in expression of Hsp90, GCR or IFNγ by CD28nullCD8+ T and NKT-like cells between COPD groups and control subjects (*p* > 0.05 for all).

**Fig. 2:**
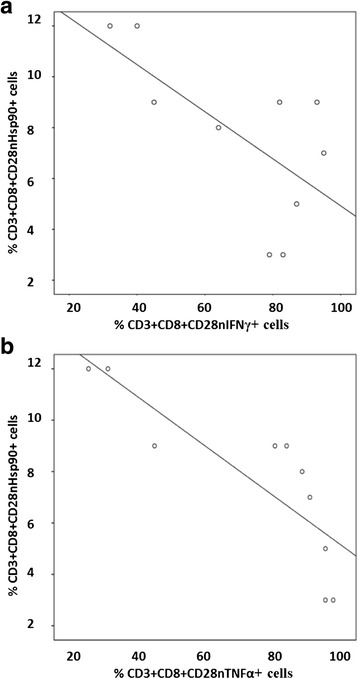
There was a significant negative correlation between the percentage of CD28nullCD8+ T cells expressing Hsp90 and producing IFNγ in COPD subjects

We found a negative correlation between loss of Hsp90 expression by CD28nullCD8+ T cells and the percentage of these cells producing IFNγ (Fig. 2a) and TNFα (Fig. 2b) in the COPD group but not the control group. There was a negative correlation between loss of Hsp90 expression by CD28nullCD8+ NKT-like cells and the percentage of these cells producing IFNγ (*R* = −.647, *P* = .039) and TNFα (*R* = −.557, *P* = .043) in the COPD group.

**Fig. 3:**
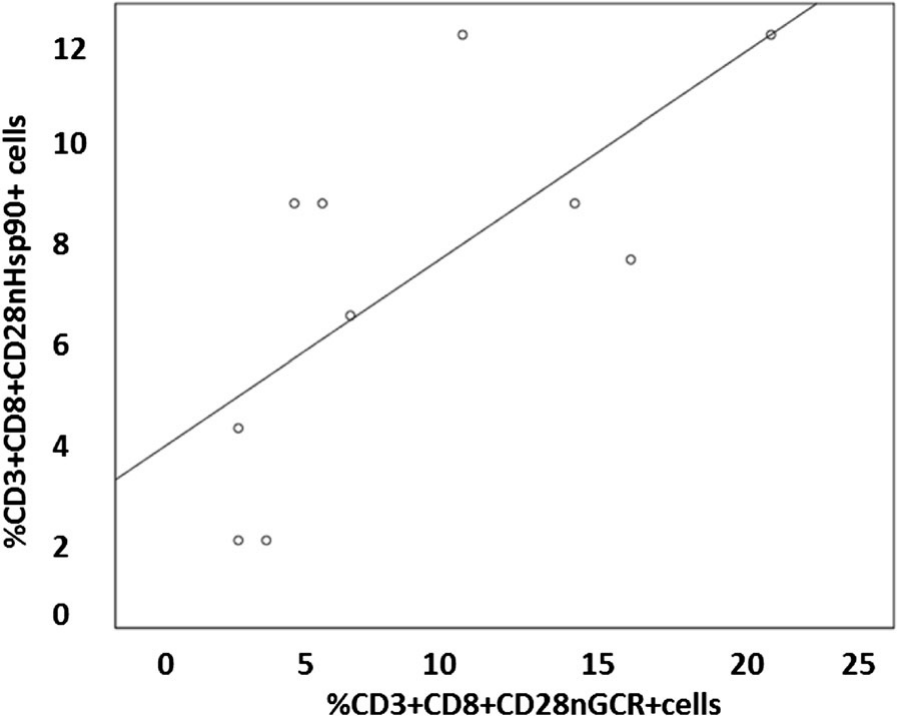
There was a significant correlation between the percentage of CD8 + CD28null T cells expressing Hsp90 and GCR in COPD subjects

A significant correlation between Hsp90 expression and GCR expression by CD28nullCD8+ T cells (Fig. 3) and NKT-like cells (data not shown) was shown, but no correlation found between any other cell subset (*p* > 0.05 for all, data not shown).

There was no significant correlation between past smoking history of COPD groups with any cell subset or Hsp90, GCR or pro-inflammatory cytokine (*p* > 0.05, data not shown).

**Fig. 4:**
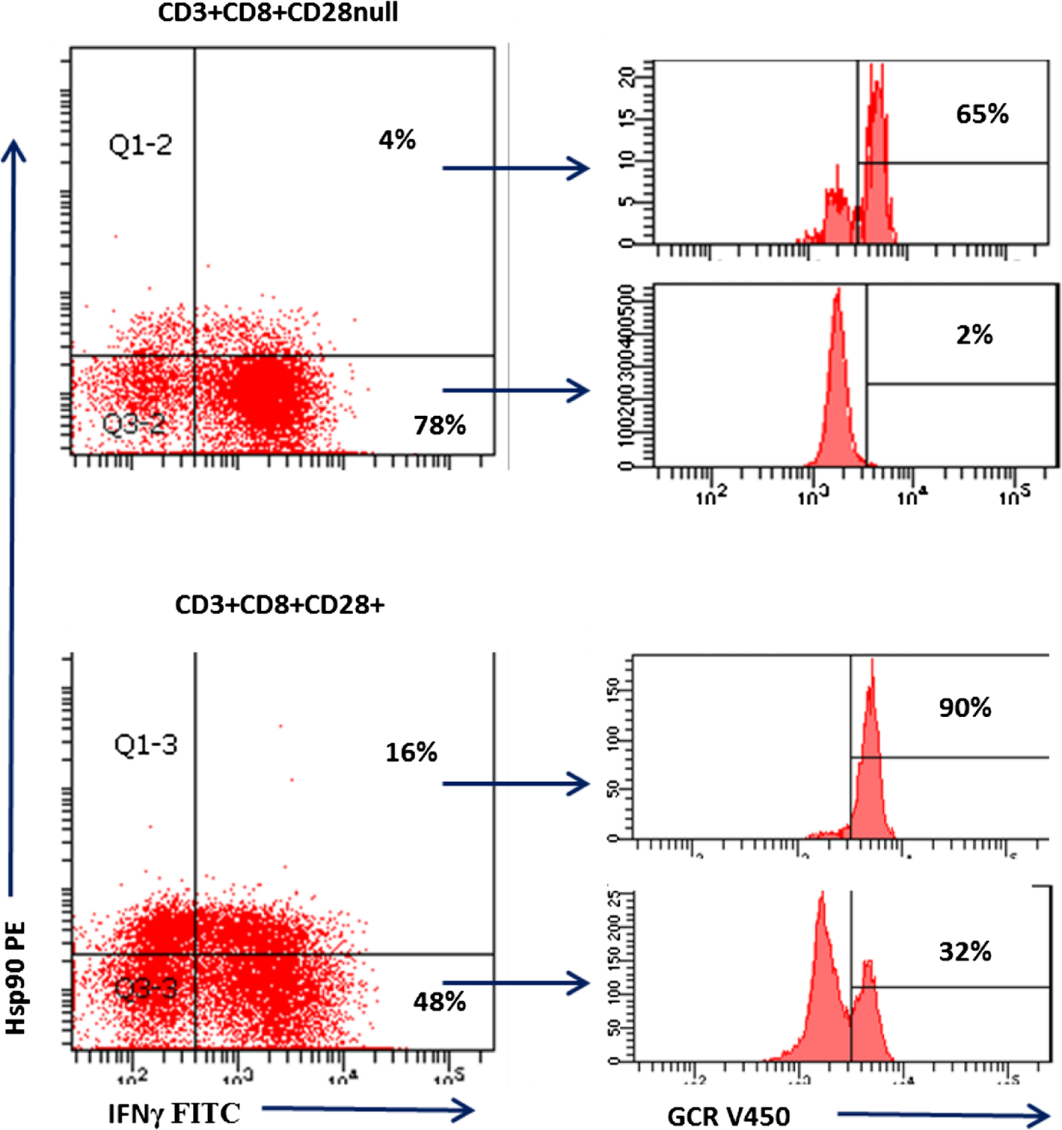
Representative plots showing expression of IFNγ and Hsp90 expression in CD3 + CD8 + CD28null (*top plots*) and CD3 + CD8 + CD28+ (*bottom plots*) cells and GCR expression in IFNγ + Hsp90+ (*upper right quadrants*) and IFNγ + Hsp90-(*bottom right quadrants*) subsets of these cells. Note the most pro-inflammatory subset (producing the most IFNγ) expresses the least GCR (2 %) and no Hsp90

Representative flow cytometry plots showing expression of IFNγ and Hsp90 expression in CD3 + CD8 + CD28null and CD3 + CD8 + CD28+ cells and GCR expression in IFNγ + Hsp90+ and IFNγ + Hsp90-subsets of these cells is shown in Fig. 4. Note the most pro-inflammatory subset (producing the most IFNγ) expresses the least GCR (2 %) and no Hsp90.

### Hsp90 expression of CD28+ and CD28null T cells by Western Blot

**Fig. 5:**
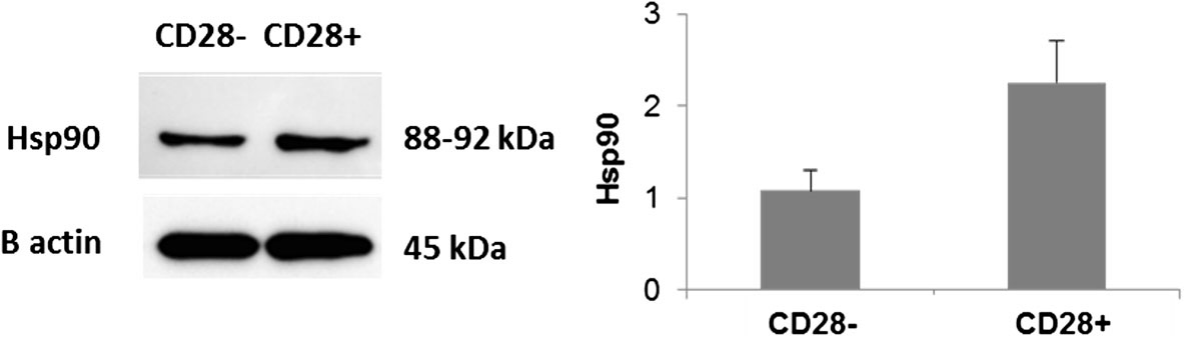
**a**. Representative Western Blot of equal numbers of sorted CD28+ and CD28null T cells, stained for Hsp90 expression. There was a decrease in the 90 kDa band corresponding to the Hsp90 in CD28null T cells compared with CD28+ T cells. **b**. Bar graph showing Hsp90 expression relative to β-actin from CD28 null (CD28−) and CD28+ T cells (mean ± sem from 3 experiments)

Equal numbers of FACS-sorted CD28+ and CD28null T cells were stained for Hsp90 expression by western analysis. There was a decrease in the 90 kD band corresponding to the Hsp90 in CD28null T cells compared with CD28+ T cells (Fig. 5a). Hsp90 expression relative to β-actin from CD28 null (CD28-) and CD28+ T cells (median ± sem from 3 experiments) is shown in Fig. 5b.

### Hsp90 is in complex with the GCR

**Fig. 6:**
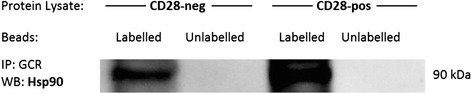
Western blot staining (WB) of equal numbers of sorted CD28+ and CD28-T cells following protein immunoprecipitation (IP) using GCR-labelled beads, and probed for Hsp90 confirmed the presence of Hsp90-GCR complexes

Protein precipitation using GCR-labelled beads, and probed for Hsp90 during western analysis confirmed the presence of Hsp90-GCR complexes (Fig. 6).

### Hsp90 expression in CD28+ and CD28null T cells by Fluorescent Microscopy

**Fig. 7:**
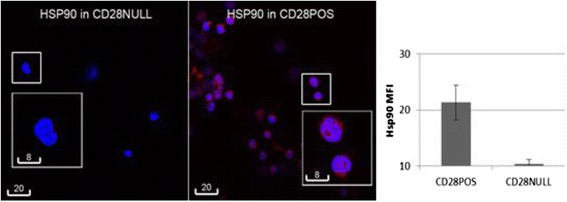
**a**. Representative laser confocal images of Hsp90 staining (*red*) in FACS-sorted CD28null (*right*) and CD28+ T cells (*left*). *Blue* was DAPI counterstaining. Scale bar = 8 μm. **b**. The bar graph depicts results of quantitative analysis by ImageJ. Experiments were repeated 3 times, showing similar results. *** *p* < 0.05

Sorted CD28+ and CD28null T cells were stained for Hsp90 expression. There was significant positive staining with Hsp90 in CD28+ T cells compared with CD28null T cells using fluorescence microscopy (Fig. 7a). Hsp90 staining was mainly located in the CD28+ T cell nucleus (Fig. 7b).

### Hsp90 expression in the cytoplasm and nucleus of CD28+ T cells compared with CD28 null T cells

**Fig. 8:**
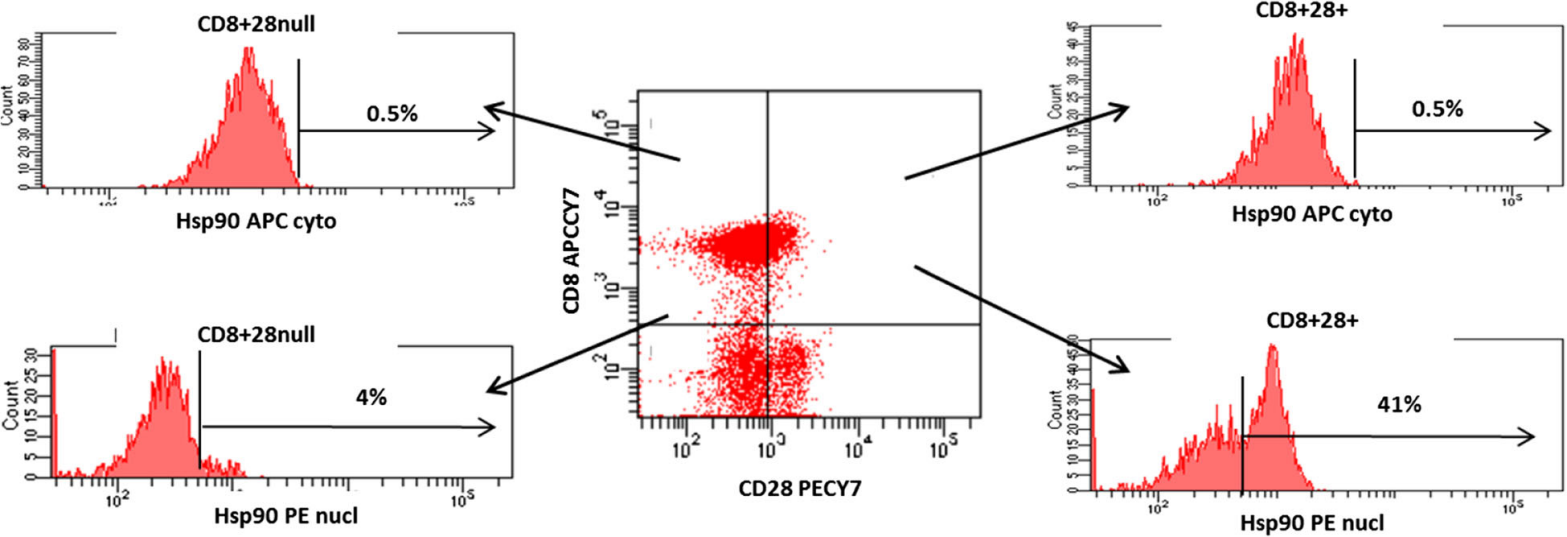
Representative flow cytometry plots showing expression of Hsp90 in CD8 + CD28null and CD8 + CD28+ T cells in the cytoplasm and nucleus following stimulation. There was a significant increase in Hsp90 expression in the nucleus of CD8 + CD28+ cells compared with CD8 + CD28null cells (*p* < 0.05 for all, from 5 experiments)

To confirm nuclear staining of Hsp90 in CD28+ T cells, differential expression of Hsp90 in the cytoplasm and nucleus of CD28+ and CD28null T and NKT-like cells was performed. There was a significant increase in Hsp90 expression in the nucleus of CD8 + CD28+ cells compared with CD8 + CD28null cells. Representative flow cytometry plots showing expression of Hsp90 in CD8 + CD28null and CD8 + CD28+ T cells in the cytoplasm and nucleus following stimulation are shown in Fig. 8.

### Correlation between Hsp90 by CD28nullCD8+ T cells and FEV1

**Fig. 9:**
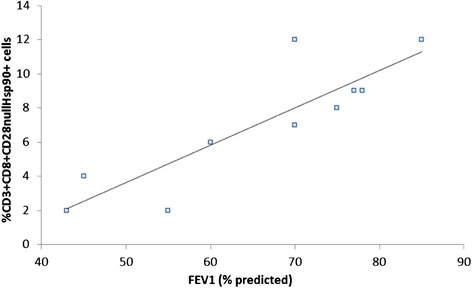
There was a significant correlation between the percentage of CD8 + CD28null T cells expressing Hsp90 and FEV1 (% predicted) in COPD subjects

There was a correlation between Hsp90 expression by CD28nullCD8+ T cells and FEV1 (% predicted) from the COPD group (Fig. 9) but no correlation between Hsp90 expression by any other lymphocyte subset with FEV1 (data not shown).

### Effect of drugs on Hsp90 and intracellular cytokine expression by CD28null CD8+ T and NKT-like cells in COPD patients

**Fig. 10:**
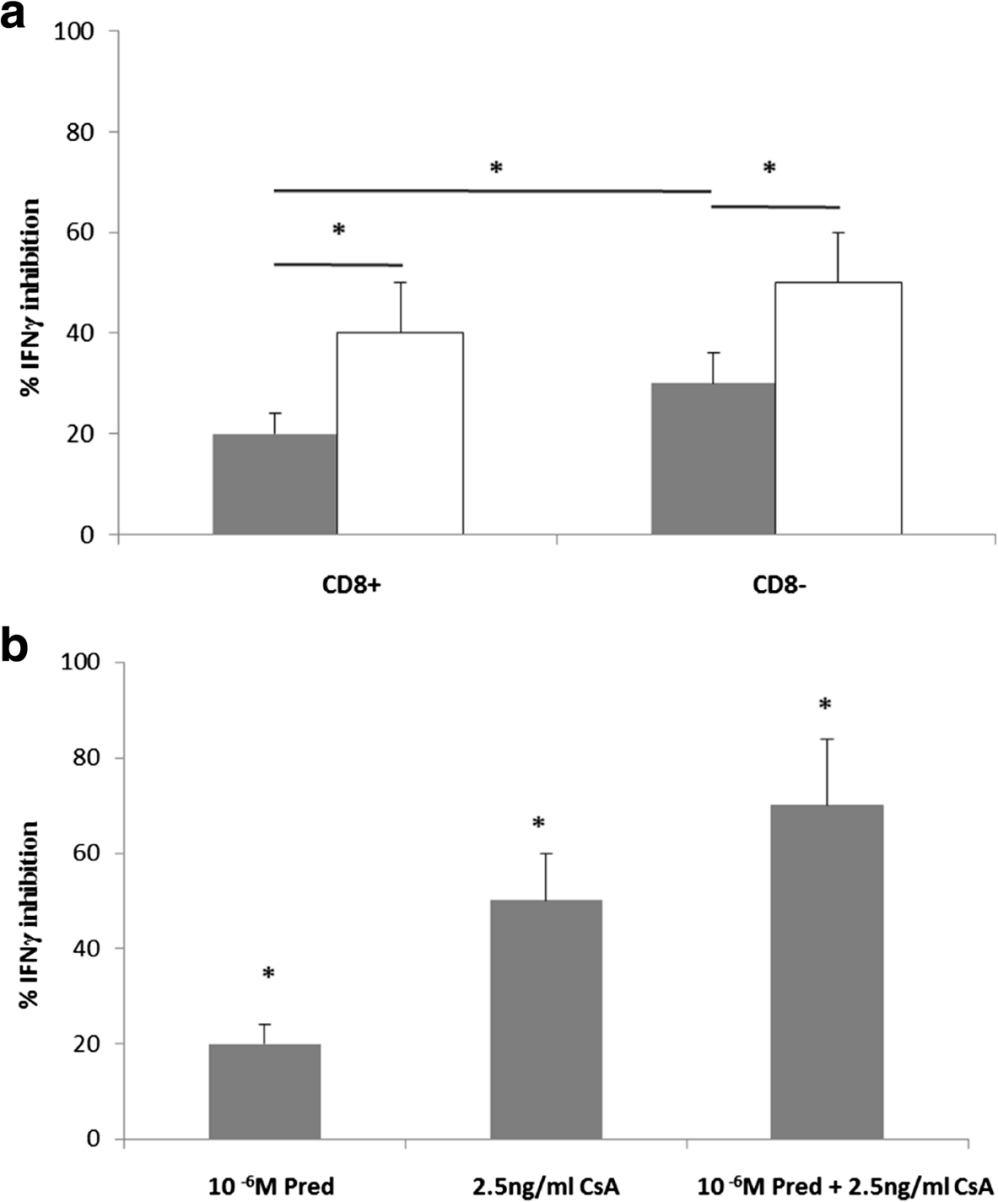
**a** Bar graph showing the inhibitory effect of 1 μM prednisolone (Pred) on IFNγ production by CD28null (*grey bars*) and CD28+ (clear bars) CD8+ and CD8-T cells compared with cultures with no drug. There was a significant inhibitory effect on CD28+ compared with CD28null cells in the presence of Pred and a significant inhibitory effect on CD28nullCD8-compared with CD28nullCD8+ cells (*n* = 5; median ± sem) (* *p* < 0.05). **b** The inhibitory effect 1 μM prednisolone ± 2.5 ng/ml cyclosporine A (CsA) on IFNγ production by CD28nullCD8+ T cells compared with cultures with no drug (*n* = 4; median ± sem) (*p < 0.05)

The effect of 1 μM prednisolone on the inhibition of IFNγ production by CD28null and CD28+ CD8+ and CD8 − T cells compared with cultures with no drug is shown in Fig. 10a. There was a significant inhibitory effect on CD28+ compared with CD28null cells in the presence of prednisolone and a significant inhibitory effect on CD28nullCD8- compared with CD28nullCD8+ cells (*n* = 5; median ± sem) (* *p* < 0.05 for all). The effect of 1 μM prednisolone (MP) ± 2.5 ng/mL CsA on the inhibition of IFNγ production by CD28nullCD8+ T cells compared with cultures with no drug is shown in Fig. 10b.

**Fig. 11:**
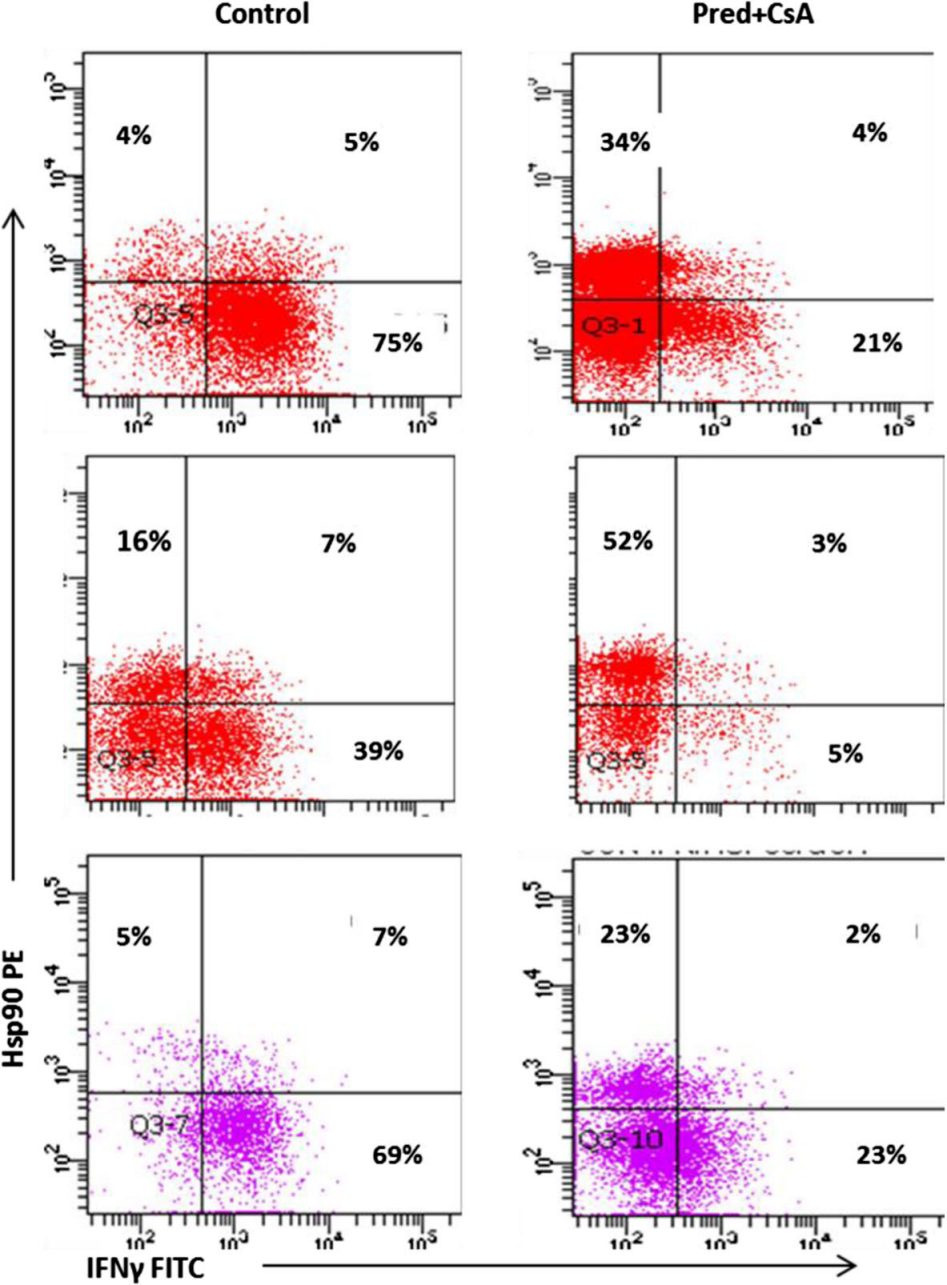
Representative dot plots showing the combined effect of 10^−6^ M prednisolone (Pred) and 2.5 ng/mL cyclosporine A (CsA) on the percentage of CD28null CD8+ T (*top plots*) and NKT-like cells (*bottom plots*) expressing Hsp90 and producing IFNγ. Note the significant increase in Hsp90 and significant decrease in IFNγ in both CD28null subsets in the presence of Pred and CsA

We also showed a significant increase in the percentage of CD28nullCD8+ T cells expressing Hsp90 in the presence of MP, CsA or a combination of both. Similar results were obtained for upregulation of Hsp90 and inhibition of IFNγ production by CD28+ and CD28nullCD8+ and CD8-NKT-like cells (ie., results were similar for all T and NKT-like subsets). Representative dot plots showing the combined effect of 1 μM prednisolone and 2.5 ng/mL CsA on the percentage of CD28nullCD8+ T and NKT-like cells expressing Hsp90 and IFNγ are shown in Fig. 11.

The presence of the Hsp90 inhibitor, 17-AAG (2 μM), negated 75 ± 12 % (median ± sem from 4 experiments) of the inhibitory effect of CsA and MP on IFNγ and TNFα by CD8+ and CD8-T and NKT-like cells.

## Discussion

This is the first study to show that lymphocyte senescence is associated with loss of molecular chaperone Hsp90 from CD8 + CD28null T and NKT-like cells. The loss of Hsp90 was shown to correlate with the cytotoxic/pro-inflammatory potential of these cells and importantly, lung function in patients with COPD. Other molecules have been reported on senescent lymphocytes indicating our present study may have underestimated the CD8+ phenotype [17].

GCR must be bound to molecular chaperones Hsp70 and Hsp90 to acquire a high-affinity steroid binding conformation, traffic to the nucleus where engagement of histone deacetylases (HDACs), particularly HDAC2, results in reduction of pro-inflammatory gene activation [17] and our findings of GCR-Hsp90 binding was confirmed using immunoprecipitation and western anaylsis. In this regard we have recently shown a loss of glucocorticoid receptor and HDAC2 expression by these senescent lymphocyte subsets [9, 10]. These findings suggest that multiple factors may be influencing steroid resistance in these cells. Interestingly, reduced Hsp90 expression has been shown to be associated with immune T cell senescence and could be mimicked by mild oxidative stress similar to that experienced in COPD [18]. Addition of exogenous rHsp90 to purified proteasome preparations could upregulate the catalytic activity of the proteasome suggesting loss of Hsp90 contributes directly to lymphocyte immune senescence [18]. In contrast, one study showed that nuclear levels of Hsp90 and Hsp70 were increased in cells isolated from induced sputum of stable COPD patients [19]; however, as most cells in induced sputum are macrophages and neutrophils, a comparison with lymphocyte subsets in our present study cannot be made. Hsp90 impairment has recently been associated with loss of CD28 molecule in lymphocytes consistent with our current study [20]. Diminished induction of Hsp90 has been observed in older subjects regardless of health status, however these differences were only evident in subjects with a mean age of 75 years and as such would probably not account for differences observed in the slightly older patient group in our study [21].

The correlation between expression of Hsp90 and GCR in the pro-inflammatory lymphocyte subsets is interesting although puzzling. Although the role of molecular chaperones in nuclear trafficking of GCR is well established [22], a possible link between Hsp90 and GCR expression is unknown and may be an anomaly of senescent lymphocytes. CD8CD28null T and NKT-like cells express the least Hsp90 and GCR [10] and recently were shown to express reduced HDAC2 [9]. Further study of HDAC2 interactions with GCR and Hsp90 in these pro-inflammatory lymphocyte subsets may also be of interest. GCR and Hsp90 were shown to be deficient in the same CD8 + CD28null T and NKT-like cells. If HDAC2 expression is also reduced in the same senescent lymphocytes, a combination of drug/s to increase expression of all three molecules may be required to completely overcome steroid resistance in these cells.

Senescent CD28null T and NKT-like cells have been shown to be more pro-inflammatory and cytotoxic than their CD28 positive counterparts [6, 9, 10], and exhibit a relative resistance to corticosteroids [10]. Increased pro-inflammatory CD8+ T cells in peripheral blood and lungs [4] and an increase in cytotoxic NKT-like and NK cells in the airways have been shown in COPD patients compared to healthy and never-smokers [7]. We have also identified increased CD28nullCD8+ cells in both current and ex-smoker COPD groups [6]. Our current study extends this data and demonstrates a negative correlation between the percentage of CD28nullCD8+ T and NKT-like cells producing pro-inflammatory cytokines IFNγ and TNFα, and the percentage expressing Hsp90. Another important extension to our studies would be to determine whether Hsp90 levels in lymphocyte subsets are altered in smokers who have not progressed to COPD and whether there is any correlation with smoking pack years. We also demonstrated that the Hsp90 deficient lymphocytes were present in the systemic circulation of COPD patients. Barnes et al. proposed a spillover of cells from the lungs into the systemic circulation [2], which suggest these Hsp90 deficient cells may have originated in the lung possibly due to increased levels of oxidative stress.

Interestingly, we showed that a loss of Hsp90 expression by CD28nullCD8+ T and NKT-like cells also occurred in healthy control subjects (Hsp90 expression was the same in CD28null T and NKT-like cells from both subject groups), although at decreased numbers compared with patients with COPD. Lymphocyte senescence and GC resistance have been described in several other inflammatory conditions, such as cardiovascular disease [23], autoimmune disease [24], arthritis [25], IBD [26], aging [27] and aging with associated inflammation in COPD [28], suggesting that these cells could potentially be involved in the onset of other inflammatory diseases in addition to COPD. Our data also highlight the importance of investigating the relative steroid resistance of the CD28null inflammatory lymphocytes with any therapeutic approaches, and the requirement for alternative ‘steroid-sparing’ anti-inflammatory therapies. We have previously shown cytotoxic/pro-inflammatory T and NKT-like cells have increased levels of drug efflux pump, Pgp-1, and the presence of very low dose CsA, a Pgp-1 inhibitor, resulted in steroid sensitivity of these cells [16]. Furthermore, we have recently shown the addition of low dose CsA increased HDAC2 levels in these lymphocyte subsets resulting in a reduction of pro-inflammatory cytokines [9]. Taken together with our current findings suggests that combined treatment with very low dose CsA and standard dose prednisolone may be a drug combination of choice to target cytotoxic/pro-inflammatory lymphocytes in patients with COPD. Our ex vivo assays to study Hsp90/GCR/HDAC2 deficient pro-inflammatory lymphocytes may identify COPD patients that would benefit from treatment with these combination of drugs. Further lymphocyte phenotyping post therapy could identify effectiveness of this therapy.

## Conclusion

Lymphocyte senescence in COPD is associated with loss of molecular chaperone in Hsp90 in CD28nullCD8+ T and NKT-like cells. This loss is associated with steroid resistant pro-inflammatory lymphocytes and lung function in COPD, thus therapies aimed at targeting pro-inflammatory senescent lymphocytes are warranted.

## Abbreviations

CD: Cluster of differentiation
COPD: Chronic obstructive pulmonary disease
CsA: Cyclosporine A
EDTA: Ethylenediaminetetraacetic acid
FEV1: Forced expiry volume in 1 s
FSC: Forward light scatter
FVC: Forced vital capacity
GCR: Glucocorticoid receptor
HDAC: Histone deacetylase
Hsp: Heat shock protein
IFNγ: Interferon gamma
NKT-like: Natural killer T cell like
PBS: Phosphate buffered saline
SSC: Side light scatter
TNFα: Tumour necrosis factor alpha
